# Association between dentition and frailty and cognitive function in community-dwelling older adults

**DOI:** 10.1186/s12877-022-03305-y

**Published:** 2022-07-25

**Authors:** Li Feng Tan, Yiong Huak Chan, Reshma A. Merchant

**Affiliations:** 1 Healthy Ageing Programme, Alexandra Hospital, 378 Alexandra Rd, Singapore, 159964 Singapore; 2 Biostatistics Unit, Yong Loo Lin School of Medicine, National University of Singapore, Singapore, Singapore; 3 Yong Loo Lin School of Medicine, National University of Singapore, Singapore, Singapore; 4 Division of Geriatric Medicine, Department of Medicine, National University Hospital, National University Health System, 1E Kent Ridge Road, Singapore, 119228 Singapore

**Keywords:** Oral health, Dentition, Frailty, Cognition

## Abstract

**Objectives:**

To evaluate dentition status amongst community-dwelling older adults and its association with frailty and cognitive impairment.

**Methodology:**

One thousand forty-seven community-dwelling older adults aged ≥65 years were surveyed in an epidemiologic population-based cohort study in Singapore between April 2015 and August 2016. Data on demographics, dentition status, chronic diseases, activities and instrumental activities on daily-living, cognition (age- and education-specific MMSE cut-offs), frailty (FRAIL scale), perceived health and functional status were collected. Multiple logistic regression was performed to examine the association between dentition, frailty and cognition.

**Results:**

Mean age of participants was 71.2 ± 5.5 years. The prevalence of denture use was 70.7% and edentulism 7.9%. Compared to edentulousness, having teeth was associated with lower odds of cognitive impairment and higher odds of being robust or pre-frail. Denture-wearers compared with edentulous persons were less likely to be male, had higher education level and more likely be robust or pre-frail.

**Conclusion and implications:**

There were significant associations between dentition status, frailty and cognition in our study where those with remining teeth and / or dentures had better overall outcomes. As oral health, frailty and cognitive impairments are all modifiable risk factors for healthy ageing, countries should consider population level screening for oral health, frailty and cognitive impairment.

## Introduction

Globally the number of older adults is increasing at an unprecedented rate and Singapore is no exception where one in four of the population will be ≥65 years old by 2030 [1]. With ageing population, the prevalence of dementia, frailty and non-communicable diseases (NCD) will increase putting a strain on health and social care resources. Maintaining oral health with ageing is crucial for healthy longevity as it is associated with better swallowing, chewing, nutrition, communication, socialisation and has positive effect on diabetes and NCD [2]. It is an important indicator of general health, well-being and quality of life [3, 4]. Studies have shown a longitudinal association between oral health [5], functional decline [6], Alzheimer’s Disease and frailty [7, 8]. Poor oral health is characterised by tooth loss and periodontitis. These are highly prevalent amongst older adults especially those with frailty and cognitive impairment [9–11]. Clinicians have limited knowledge and guidance on appropriate dental treatment for complex older adults and similarly, there is limited integration of frailty and cognition assessment in dental practice.

Frailty is a dynamic state, affecting multiple physiological system and refers to a state of increased vulnerability to stressors resulting in functional decline, falls, increased morbidity and mortality. The prevalence of frailty in medical outpatient clinic locally is 27% and community 6% [12, 13]. Frailty is reversible with targeted interventions such as nutrition, exercise, polypharmacy and oral health management [14, 15]. Number of remaining teeth is a known predictor of frailty and frail edentulous older adults also have reduced maximum bite force and impaired mastication escalating the downward spiral to disability [16, 17]. Other factors associated with poor oral health include cognitive impairment and/or dementia, smoking and body mass index. Frailty, cognitive impairment and poor oral health share common risk factors including poor nutrition, chronic inflammation and reduced socialisation.

Studies have shown that older adults with 20 or more teeth have overall reduced prevalence of frailty [18, 19] and cognitive impairment [20] compared with edentulous older adults. The ‘Healthy Japan 21’ strategy recommends on keeping at least 20 or more teeth by the age of 80 years old encouraging collaborations between health authorities, dental associations and domiciliary dental services [21]. Singapore has introduced Project Silver Screen, a nation-wide programme for ≥60 years old to screen on vision, hearing and oral health. However, there is limited information on risk factors and personalised goal targeted stage-specific dental recommendations. Our study aimed to evaluate 1) the dentition status and 2) risk factors and association of dentition status with frailty and cognitive impairment in community dwelling older adults.

## Methods

### Study population

The Healthy Older People Everyday (HOPE) study is an epidemiologic population-based study involving a sample of 1051 community-dwelling older adults aged 65 years and older from a defined geographical area in the Northwest region of Singapore. Baseline data were collected between April 2015 and August 2016. Oral health data was available for 1047 older adults. This study was part of the larger Singapore Population Health Studies (SPHS) cohort in the constituency of Bukit Panjang [22]. An invitation letter was sent to all household units in the public housing estates of the constituency, banners and posters were also put up at public places by the local Constituency Office and word about the health survey was disseminated to the community through their network of grassroots volunteers. Non-responders were approached by trained interviewers in their own home and were also invited to participate.

### Consent and ethics approval

Written consent was obtained prior to interviews and interviews were conducted at the participants’ homes or at a place of the participant’s preference [22, 23]. The research was approved by the National Healthcare Group, Singapore Institutional Review Board [Ref: 2019/00017], accessible at https://research.nhg.com.sg.

### Demographic data and dentition status

Interview questions were administered by trained research assistant and data on demographics including education level, dentition status, chronic diseases, activities and instrumental activities on daily-living, cognition, frailty and perceived health were collected. Specifically, dentition status was part of a self-reported questionnaire and was classified as having natural teeth but no dentures, denture-wearing and edentulous.

### Assessment of cognitive function, frailty and covariates

Cognitive function was assessed using the Mini Mental State Exam (MMSE) administered by trained research assistants [24]. We adopted age and education-specific cut-off points for the definition of cognitive impairment [25]. Cut-off points for classifying as cognitively impaired in ≥75 years old was 17/18 for those with Primary School education, 22/23 for secondary school or higher education. In < 75 years, 20/21 for those with primary school education, and 23/24 for those with secondary school or higher education [25].

Polypharmacy was defined as the presence of five or more long-term medications. Functional status was assessed using the Lawton IADL Scale and Barthel Index [26, 27]. Frailty was assessed using the 5-item FRAIL scale (Fatigue, Resistance, Aerobic, Illness, and Loss of Weight) which has been validated in Asian countries and locally [13, 28, 29]. The FRAIL scale has a maximal score of 5, participants with a score of 3 to 5 are categorised as frail, 1-2 as pre-frail, and zero as being robust. Perceived health was recorded based on the EuroQol vertical visual analog scale. Multi-morbidity was defined as presence of 2 or more of the following comorbidities: hypertension, hyperlipidemia, diabetes mellitus, heart disease, cancer, stroke, and lung disease.

Anthropometric measurements, including weight, height, and body mass index (BMI) were measured. Handgrip strength (HGS) and the Timed Up and Go (TUG) tests were performed as part of functional screening. HGS was measured using hand dynamometers (Takei A5401, Japan) in kilograms (kg). Three readings were taken for each hand for a total of 6 readings. However, only the maximum grip strength reading for the dominant hand was used in analyses.

### Statistical analysis

Mean and standard deviations were calculated for continuous variables, while frequencies and percentages were calculated for categorical variables. Univariate analysis was performed using chi-square test for categorical variables and one-way analysis of covariance or Kruskal Wallis for continuous variables to assess for differences amongst dentition status and sociodemographic, medical, and cognitive variables. Multiple logistic regression was performed to identify independent predictors (demographic, cognitive function, frailty, grip strength, TUG, multimorbidity, IADL, ADL, falls, fatigued and weight loss) of dentition status in older adults. Statistical significance was determined by using the cut-off *p* value of 0.05 at the 2-tailed level. All statistical analyses were performed using IBM SPSS, version 26.0.

## Results

### Baseline characteristics

A total of 1047 older adults aged 65 and above completed the screening with available data on oral health. Basic demographics of the cohort are shown in Table 1. The mean age of participants was 71.2 ± 5.5 years, 57.2% were female, while 79.9% were of Chinese ethnicity. The mean years of education was 6.0 ± 4.4. Amongst the study population, 56.7% were robust, 37.1% pre-frail and 6.2% frail. The prevalence of denture use was 70.7%, edentulism 7.9 and 21.3% reported having own teeth without dentures.

**Table 1:** Baseline demographics and characteristics according to dentition status

	All *N* = 1047	Natural teeth, no dentures 224 (21.3%)	Denture-wearing, with / without natural teeth) 740 (70.7%)	No Teeth 83 (7.9%)	*p* value
Age (mean)	71.2 ± 5.5	70.3 ± 4.9^2^	71.3 ± 5.4^3^	72.9 ± 6.8^2 3^	**0.001**
Gender					
Male	448 (42.8)	121 (54.0)	290 (39.2)	37 (44.6)	**< 0.001**
Female	599 (57.2)	103 (46.0)	450 (60.8)	46 (55.4)	
Education (years)	6.0 ± 4.4	7.7 ± 4.6	5.8 ± 4.3	3.3 ± 3.6	**< 0.001**
MMSE	26.2 ± 4.4	26.6 ± 4.5^2^	26.2 ± 4.2^3^	24.5 ± 5.4^2 3^	**0.001**
Cognitive Impairment^a^	117 (11.2)	14 (6.3)^1^ ^2^	87 (11.8)^1^ ^3^	16 (19.3)^2^ ^3^	
EQ-VAS	80.2 ± 15.6	81.3 ± 15.3^2^	80.3 ± 15.0^3^	75.8 ± 20.6^2 3^	**0.019**
Grip strength (kg)	21.8 ± 7.0	23.3 ± 7.2^1^	21.4 ± 6.9^1^	21.6 ± 5.7	**0.004**
TUG, completion time	11.3 ± 4.0	11.1 ± 3.7	11.3 ± 4.0	12.5 ± 4.7	0.109
BMI	24.4 ± 4.1	24.8 ± 4.5	24.3 ± 3.9	24.4 ± 4.3	0.283
Waist circumference	87.2 ± 10.8	87.8 ± 11.9	86.9 ± 10.2	89.0 ± 12.8	0.327
Multimorbidity	552 (52.7)	113 (50.4)	395 (53.4)	44 (53.0)	
Ethnicity					
Chinese	837 (79.9)	134 (16.0)	644 (76.9)	59 (71.1)	**< 0.001**
Malay	73 (7.0)	31 (13.8)	34 (45.9)	8 (9.6)	
Indian	69 (6.6)	29 (12.9)	32 (43.2)	8 (9.6)	
Others	68 (6.5)	30 (13.4)	30 (40.5)	8 (9.6)	
FRAIL					
Robust	594 (56.7)	140 (62.5)	415 (56.1)	39 (47.0)	**0.001**
Pre-frail	388 (37.1)	76 (33.9)	281 (38.0)	31 (37.3)	
Frail	65 (6.2)	8 (3.6)	44 (5.9)	13 (15.7)	
≥ 1 IADL Impairment	427 40.8)	94 (42.0)	288 (38.9)	45 (54.2)	**0.025**
≥ 1 ADL Impairment	195 (18.6)	45 (20.1)	129 (17.4)	21 (25.3)	0.178
≥ 2 falls	96 (9.2)	23 (10.3)	65 (8.8)	8 (9.6)	0.787
Fatigued	265 (25.4)	43 (19.2)^1^	206 (27.8)^1^	16 (19.3)	**0.015**
Weight loss > 5% over 6 months	141 (13.5)	32 (14.3)	96 (13.0)	13 (15.7)	0.717
Smoking history	302 (28.8)	74 (33.0)	205 (27.7)	23 (27.7)	0.295

Values are expressed as mean ± SD (standard deviation) or n (%). Abbreviations: *MMSE* Mini-mental state examination, *EQ-VAS* European Quality of Life Visual Analogue Scale, *TUG* Timed up and go, *BMI* Body mass index ^a^Adjusted for education and age

^1^natural teeth vs denture-wearing, ^2^natural teeth vs edentulous, ^3^denture-wearing vs edentulous, *p <* 0.05

### Univariate analysis

On univariate analysis (Table 1), edentulous older adults compared with those with own teeth were older (72.9 ± 6.8 vs 71.2 ± 5.5, *p =* 0.001), had fewer years of education (3.3 ± 3.6 vs 7.7 ± 4.6, *p* <  0.001), had lower MMSE scores (24.5 ± 5.4 vs 26.6 ± 4.5, *p =* 0.001), had lower self-reported perceived health (75.8 ± 20.6 vs 81.3 ± 15.3, *p =* 0.019), higher prevalence of frailty (15.7% vs 3.6%, *p =* 0.001) and greater impairment in at least one IADL impairment (54.2% vs 10.5%, *p =* 0.025).

For edentulous older adults compared with those with dentures similarly had lower education levels (3.3 ± 3.6 vs 5.8 ± 4.3, *p* <  0.001), lower MMSE scores (24.5 ± 5.4 vs 26.2 ± 4.2, *p =* 0.001), higher prevalence of frailty (15.7% vs 5.9%), lower perceived health (75.8 ± 20.6 vs 80.3 ± 15.0, *p* = 0.019) and were less fatigued (19.5% vs 28.0%, *p =* 0.015).

### Multivariate Analysis

On multivariate analysis, when comparing older adults with natural teeth vs edentulous older adults (Table 2), having teeth was associated with a lower odds of being cognitively impaired (OR 0.14 (0.03 – 0.75), *p =* 0.022) and higher odds of being robust (OR 30.0 (4.0 - 223), *p =* 0.001) or pre-frail (OR 13.7 (2.1 – 91), *p =* 0.007). When comparing those with dentures vs edentulous older adults (Table 2), denture wearing was associated with lower odds amongst males (OR 0.38 (0.17 – 0.86), *p =* 0.020), more years of education (OR 0.91 (0.84 – 0.98), *p =* 0.015) and higher odds amongst the robust (OR 7.5 (2.1 – 2.03), *p =* 0.002) and pre-frail (OR 6.1 (1.8 - 20.7), *p =* 0.003).

**Table 2:** Multivariate analysis comparing characteristics of natural teeth & denture-wearing vs edentulous older adults

	Edentulous^ (reference category)	Natural teeth, no dentures				Denture-wearing, with / without natural teeth			
		Unadjusted		Adjusted		Unadjusted		Adjusted	
		OR (95% CI)	*p* value	OR (95% CI)	*p* value	OR (95% CI)	*p* value	OR (95% CI)	*p* value
Age	72.9 ± 6.8	0.92 (0.88-0.96)	**< 0.001**	1.03 (0.92-1.2)	0.597	0.95 (0.92-0.99)	**0.011**	1.03 (0.95-1.1)	0.474
Male	37 (44.6)	1.5 (0.88-2.4)	0.143	0.87 (0.34-2.2)	0.760	0.80 (0.51-1.3)	0.342	0.38 (0.17-0.86)	**0.020**
Education (years)	3.3 ± 3.6	0.93 (0.86-0.99)	**0.047**	0.99 (0.77-1.04)	0.163	0.95 (0.90-1.01)	0.062	0.91 (0.84-0.98)	**0.015**
Cognitive impairment	16 (19.3)	0.28 (0.13-0.60)	**0.001**	0.14 (0.03-0.75)	**0.022**	0.56 (0.31-1.01)	0.052	0.29 (0.10-0.84)	**0.023**
EQ-VAS	75.8 ± 20.6	1.03 (1.01-1.05)	**< 0.001**	1.02 (0.99-1.1)	0.145	1.02 (1.01-1.04)	**0.001**	1.02 (0.99-1.04)	0.143
Grip strength	21.6 ± 5.7	1.04 (0.99-1.09)	0.112	1.04 (0.97-1.1)	0.302	0.99 (0.96-1.04)	0.846	1.02 (0.95-1.08)	0.606
TUG	12.5 ± 4.7	0.95 (0.87-1.03)	0.221	1.2 (0.98-1.4)	0.083	0.99 (0.93-1.06)	0.803	1.11 (1.0-1.3)	0.053
BMI	24.4 ± 4.3	1.02 (0.96-1.1)	0.501	1.1 (0.95-1.2)	0.278	0.99 (0.93-1.07)	0.862	1.02 (0.93-1.2)	0.640
Multimorbidity	89.0 ± 12.8	0.90 (0.55-1.5)	0.690	1.3 (0.49-3.3)	0.622	1.01 90.64-1.6)	0.949	1.6 (0.73-3.6)	0.236
Ethnicity			0.352		0.761		**0.002**		0.096
Chinese	44 (53.0)	1.0	–	1.0	–	1.0	–	1.0	–
Malay	59 (71.1)	1.7 (0.74-3.9)	0.210	1.3 (0.28-6.0)	0.745	0.39 (0.17-0.88)	**0.023**^**a**^	0.31 (0.85-1.3)	0.107
Indian	8 (9.6)	1.6 (0.69-3.7)	0.276	1.4 (0.31-6.3)	0.665	0.37 (0.16-0.83)	**0.016**^**a**^	0.31 (0.08-1.2)	0.099
Others	8 (9.6)	1.7 (0.71-3.8)	0.241	2.5 (0.43-15.3)	0.306	0.34 (0.15-0.78)	**0.011**^**a**^	0.33 (0.09-1.3)	0.103
FRAIL	8 (9.6)		**0.001**		**0.004**		**0.005**		**0.005**
Robust	39 (47.0)	5.8 (2.2-15.1)	**< 0.001**	30.0 (4.0-223)	**0.001**	3.1 (1.6-6.3)	**0.001**	7.5 (2.1-2.03)	**0.002**
Pre-Frail	31 (37.3)	4.0 (1.5-10.6)	**0.005**	13.7 (2.1-91)	**0.007**	2.7 (1.3-5.5)	**0.007**	6.1 (1.8-20.7)	**0.003**
Frail	13 (15.7)	1.0		1.0		1.0		1.0	1.0
≥ 1 IADL Impairment	45 (54.2)	0.61 (0.37-1.01)	0.058	0.46 (0.17-1.2)	0.123	0.54 (0.34-0.85)	**0.008**	0.47 (0.21-1.04)	0.063
≥ 1 ADL Impairment	21 (25.3)	0.74 (0.4-1.3)	0.325	2.0 (0.52-2.7)	0.309	0.62 (0.37-1.06)	0.080	1.2 (0.38-3.7)	0.765
≥ 2 falls	8 (9.6)	1.07 (0.46-2.5)	0.871	0.79 (0.23-2.7)	0.709	0.90 (0.42-2.0)	0.795	0.65 (0.22-1.9)	0.427
fatigued	16 (19.3)	1.01 (0.53-1.9)	0.964	1.2 (0.36-3.7)	0.808	0.62 (0.35-1.1)	0.105	0.77 (0.31-1.9)	0.566
Weight loss > 5% over 6 months	13 (15.7)	1.1 (0.56-2.3)	0.743	0.68 (0.18-2.5)	0.565	1.3 (0.67-2.4)	0.475	0.61 (0.19-2.0)	0.414
Smoking history	23 (27.7)	1.3 (0.74-2.2)	0.373	1.2 (0.40-3.7)	0.734	1.0 (0.60-1.6)	1.0	1.4 (0.55-3.5)	0.497

Values are expressed as mean ± SD (standard deviation) or n (%). Abbreviations: *EQ-VAS* European Quality of Life Visual Analogue Scale, *TUG* Timed up and go, *BMI* Body mass index

^**a**^Adjusted for education and age

## Discussion

In this study, we investigated sociodemographic, physical and cognitive factors associated with dentition status in community dwelling older adults. Our results suggest that fewer years education was associated with a greater likelihood of being edentulous in later life. There was a higher prevalence of cognitive impairment and physical frailty amongst edentulous older adults which remains significant after adjusting for covariates. Denture-wearing was more common in women than men and amongst the robust and pre-frail when compared with the edentulous group (Table 2). After adjustment, cognitive impairment was significantly less prevalent in those with natural teeth and dentures compared with edentulous older adults. Similarly, those with own teeth or dentures were more likely to be robust or pre-frail.

Multimorbidity was prevalent in more than half of our study participants and dental professionals often deal with complex older adults. Age, cognitive impairment and frailty can be barriers to accessing timely and high-quality dental care. In addition, many frail older adults who visit dentist get routine care rather than tailored stage-specific dental treatment. There is currently an underappreciation of improved oral health and dentition as a modifiable risk factor for the development of dementia and frailty in the medical literature [17, 30, 31].

### Cognition and dentition status

Edentulous participants had significantly lower MMSE scores in our study. Association of tooth loss and periodontal disease with cognitive decline is an emerging area of research with bidirectional relationship [20, 32–36]. While direct causation has not been fully established, proposed mechanisms include poor nutritional status due to decreased quality of food intake from tooth loss affecting intake of relevant nutrient and vitamins important for the brain [31], impaired masticatory function due to tooth loss where masticatory capacity is associated with greater cerebral blood flow and oxygenation and chronic inflammation with recurrent bacterial invasion from periodontal disease which is highly prevalent in those with Alzheimer’s disease [20, 37, 38]. Chen et al. showed that a 10-year exposure to chronic periodontitis was associated with almost two-fold greater risk of Alzheimer’s disease [39]. Periodontitis is associated with both tooth loss and non-communicable diseases which are risk factors for dementia [37, 40]. Persons with dementia in turn may have reduce attention to oral hygiene which may further affect their oral health status [38]. Prior studies have shown partial retention of own teeth and timely replacement of missing teeth with dentures can attenuate cognitive decline but not in those with complete tooth loss [20, 32, 41, 42]. A possible explanation for this is that the masticatory efficiency with normal occlusion of denture wearers is as effective as those with full dentition, and thus increasing cerebral blood flow, oxygen level and cortical activation [32]. As such, preserving as many natural teeth as possible and timely rehabilitation of the missing teeth with dentures are important measures to delay cognitive decline amongst at risk older adults.

### Physical frailty and dentition status

Prevalence of frailty in edentulous participants was significantly higher compared with those with own teeth or dentures further supporting the growing body of evidence on oral health as a predictor and marker of frailty [7, 43]. The association between dentition status and frailty was even stronger after adjusting for confounding factors suggesting that frailty is indeed a strong predictor independent of education, comorbidities or other physical factors. Oral health and the pathogenesis of frailty is likely multidimensional and includes effects on chronic inflammatory pathways, masticatory function and nutritional status, physical and cognitive decline and even social behaviour [3, 44]. Similar to cognitive impairment, there is bidirectional relationship between frailty and oral health. In a recent population-based case-control study, those with edentulism and poor oral health were at increased risk of developing frailty over a 12 month period [45]. In another recent study by Albani et al., dry mouth, difficulty swallowing, difficulty eating and tooth loss were associated with slow gait speed [46]. Frailty is associated with polypharmacy, which may further exacerbate the dry mouth problem. Frail older adults may abandon visits to dentist due to mobility limitation and may reduce toothbrushing frequency due to fatigue or dexterity problems [47]. Further studies on the complex relationship between oral health, oral frailty and physical and cognitive frailty are needed to further elucidate the associations and underlying mechanisms.

### Other variables

Own teeth and dentures were associated with better perceived health. Prosthodontic treatment and own teeth has positive effect on perceived health [48]. Some factors were not found to be significantly associated with dentition status such as smoking status, weight loss, BMI or multimorbidity amongst others (Table 1). There could be several possible reasons for this. The study population were community-dwelling older adults with the majority being robust (56.7%) and pre-frail (37.1%). Only 6.2% were frail. It is likely that for dentition to have a significant measurable impact on anthropometric measures and BMI that participants would be further down the frailty trajectory. The effects of poor oral health on nutrition and weight loss were mostly seen in frail older adults and those in residential care facilities [49]. In an affluent society like Singapore, the effects of dentition status on anthropometric measurements could have been attenuated with oral nutritional supplements, dietetics intervention and dental aids such as dentures. The high prevalence of dentures (70.7% vs 7.9% edentulous) suggests a high rate of tooth loss but also a high prevalence of dental access and intervention.

### Strengths and limitations

The largest strength of our study is population level data, large sample size, inclusion of various established and potential covariates but this study has several limitations which warrants mention. Dentition status was self-reported and objective oral examination was not conducted by trained personnel. In addition, we did not collect data on numbers of remaining teeth. Tooth loss and dentition status are only one aspect of oral frailty. Our study did not examine other aspects of oral health such as presence of periodontal disease, chewing ability, tongue pressure or sites and causes of tooth loss. As a cross sectional study, causal inference is limited. Our study did not include socioeconomic data as majority of the older adults in our study were retired and we did not have information on the ownership of their homes.

### Future directions

Oral health is both a marker and predictor of frailty and cognitive impairment. Oral health is a modifiable risk factor which is often neglected in clinical practice. Clinical practice standards to guide management of complex older adults with personalised stage-specific targeted therapy in dental practice is an urgent unmet need and it should be every country’s priority to integrate oral health in routine clinical practice management and primary care. Every physician should screen for oral health issues opportunistically, and dental professionals should screen for frailty and cognitive impairment with appropriate management. There are various short screening tools for frailty and cognitive impairment with assisted management pathway such as The Rapid Geriatric Assessment which also available in the Itunes store and can be administered by dental professionals or assistants [50, 51].

## Conclusions and implications

Significant association between dentition status, frailty, quality of life and cognition were found in our study where those with remining teeth and / or dentures had better overall outcomes. As oral health, frailty and cognitive impairments are all modifiable risk factors for healthy ageing, it should be every country’s priority to have population level upstream screening for oral health, frailty and cognitive impairment.

## Data Availability

The datasets generated and/or analysed during the current study are not publicly available due to data protection regulations in Singapore but are available from the corresponding author on reasonable request.
